# Capping nanoparticles with graphene quantum dots for enhanced thermoelectric performance†
†Electronic supplementary information (ESI) available. See DOI: 10.1039/c5sc00910c


**DOI:** 10.1039/c5sc00910c

**Published:** 2015-04-13

**Authors:** Yuantong Liang, Chenguang Lu, Defang Ding, Man Zhao, Dawei Wang, Chao Hu, Jieshan Qiu, Gang Xie, Zhiyong Tang

**Affiliations:** a CAS Key Lab for Nanosystem and Hierarchy Fabrication , National Center for Nanoscience and Technology , Beijing , 100190 , China . Email: lucg@nanoctr.cn ; Email: zytang@nanoctr.cn; b Key Laboratory of Synthesis and Natural Functional Molecular Chemistry of Ministry of Education , College of Chemistry & Materials Science , Northwest University , Xi'an , 710069 , China . Email: xiegang@nwu.edu.cn; c Carbon Research Laboratory , Center for Nano Materials and Science , State Key Laboratory of Fine Chemicals , School of Chemical Engineering and Key Laboratory for Micro/Nano Technology of Liaoning Province , Dalian University of Technology , Dalian 116024 , China

## Abstract

Graphene quantum dots (GQDs) are shown to serve as phase transfer agents to transfer various types of nanoparticles (NPs) from non-polar to polar solvents. Thorough characterization of the NPs proves complete native ligand exchange. Pellets of this GQD–NP composite show that the GQDs limit the crystal size during spark plasma sintering, yielding enhanced thermoelectric performance compared with NPs exchanged with inorganic ions. A photoluminescence study of the GQD–NP composite also suggests energy transfer from GQDs to NPs.

## Abstract 2

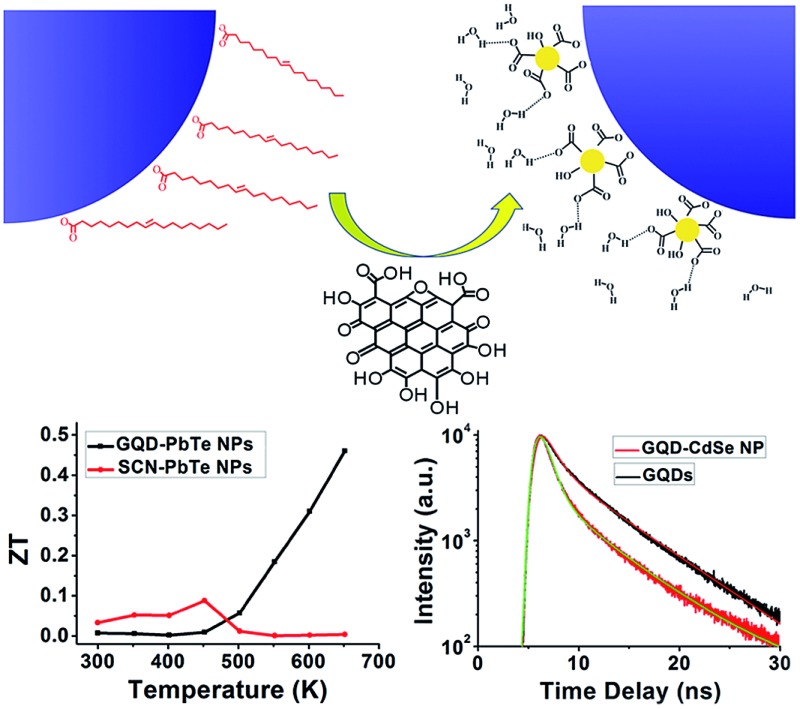
The general capability of graphene quantum dots to serve as capping ligands exchanging native organic stabilizers for various types of semiconductor nanoparticles affords the opportunity to engineer functional nanocomposites with remarkable thermoelectric properties.

## Introduction

Inorganic nanoparticles (NPs) have many unique properties that have attracted much attention since their introduction.^1^ However, the most versatile wet-chemistry synthetic methods for producing NPs inevitably coat them with long chain organic ligands, which insulate the NPs from each other and their environment. Removal of such organic coatings thus becomes a key challenge for incorporating NPs into devices as functional parts. Many agents have been proposed to replace the native ligands, including inorganic anions,^2^–^4^ chalcogenide complexes,^5^,^6^ NOBF_4_,^7^ Meerwein’s salt,^8^ formic acid,^9^,^10^ thiolate ligands,^11^–^14^ and polymers.^15^,^16^ These agents effectively strip the NPs of their native ligands and bring them closer together, improving electrical conductivity and energy transfer. However, these native ligand exchangers, except for metal chalcogenide complexes and polymers, are mostly small chemical species and serve as stabilizing agents only.

Recent advances in nanocomposite materials allow for a scalable methodology to generate multifunctional materials with properties stemming from both their individual components and, more interestingly, their synergistic interactions.^17^–^22^ When incorporating NPs in such nanocomposites, for the sake of property versatility one would desire a ligand with capabilities beyond just colloidal stabilization, *i.e.* a ligand with functionalities. Herein, we present the use of graphene quantum dots (GQDs) as ligands to stabilize nanoparticles.

GQDs can be viewed as a derivative of the extensively studied two-dimensional material graphene.^23^–^27^ They are a class of nanometer-sized graphitic sheets with abundant edge functional groups.^28^,^29^ Their size-related band gap and photoluminescence (PL) properties have permitted their application in bio-labeling.^29^,^30^

**Fig. 1:**
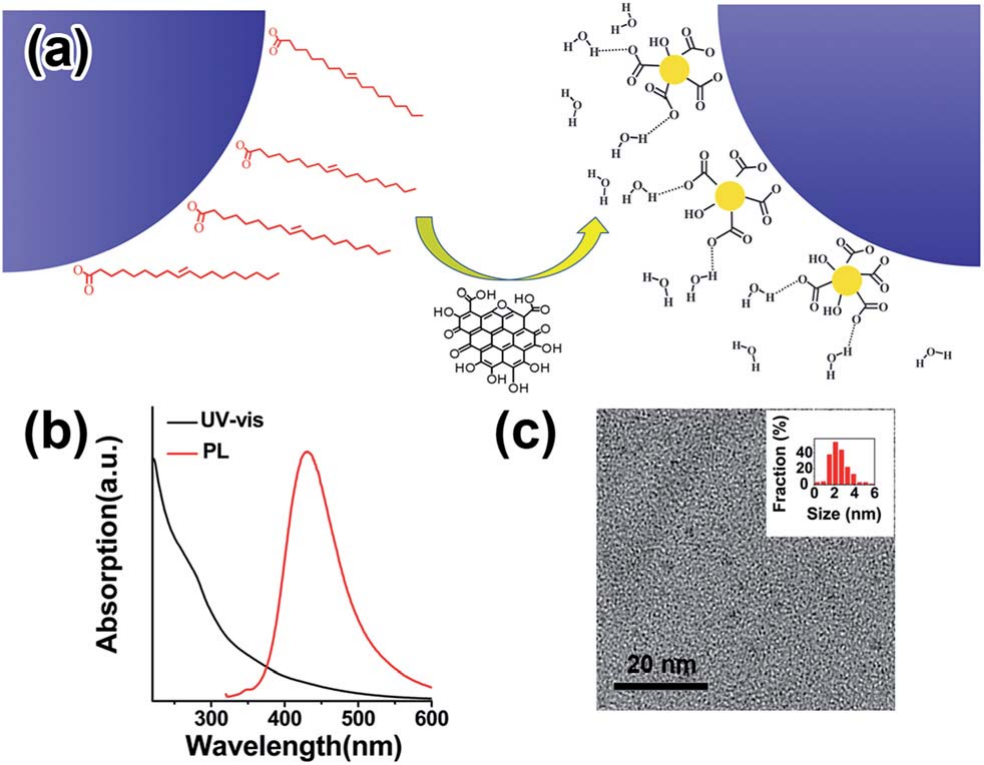
(a) Schematic drawing of how GQDs may be used as capping ligands to replace native oleic acid ligands. (b) UV-vis and photoluminescence spectra of GQDs. (c) TEM image of GQDs; the inset presents the histogram of the size distribution of GQDs.

Recent incorporation of GQDs into nanocomposite materials also offers the opportunity to take advantage of their unique charge carrier extraction capability for better solar cell efficiency.^31^,^32^ In this communication, we demonstrate that GQDs can be used directly as a native ligand exchanger and to also stabilize NPs in polar solvents (Fig. 1a).^33^ We further show that, when the composite is made into a pellet by spark plasma sintering (SPS), the fusing of NPs is lessened and significantly enhanced thermoelectric performance is achieved, most likely due to the preservation of quantum confinement and carrier energy filtering.^34^,^35^ This novel type of GQD ligand is thus advantageous over conventional molecular ligands when high temperature ligand stability is needed.

## Results and discussion

Inorganic NPs were made following well-developed wet-chemistry methods.^36^–^39^ For GQDs synthesis, graphene oxide (GO) from natural graphite powder was first prepared by a modified Hummer's method,^40^ and a hydrothermal method was then adopted to cut the GO into small pieces of GQDs^41^,^42^ (details in the ESI†). The as-synthesized GQDs are highly luminescent with a PL peak at 440 nm (Fig. 1b), while transmission electron microscopy (TEM) imaging shows the GQDs have a uniform size of around 2.5 nm (Fig. 1c), proving them to be of high quality. The ligand exchange processes were carried out in a nitrogen-filled glovebox. For a typical ligand exchange, 3 mL of NPs in toluene solution was added to 3 mL of GQDs in formamide and vigorously stirred for several hours. After complete phase transfer, the toluene phase was discarded, and the formamide phase was washed three times with fresh toluene. The resulting GQD–NPs were precipitated by acetone and finally redispersed in DMF or DMSO.

**Fig. 2:**
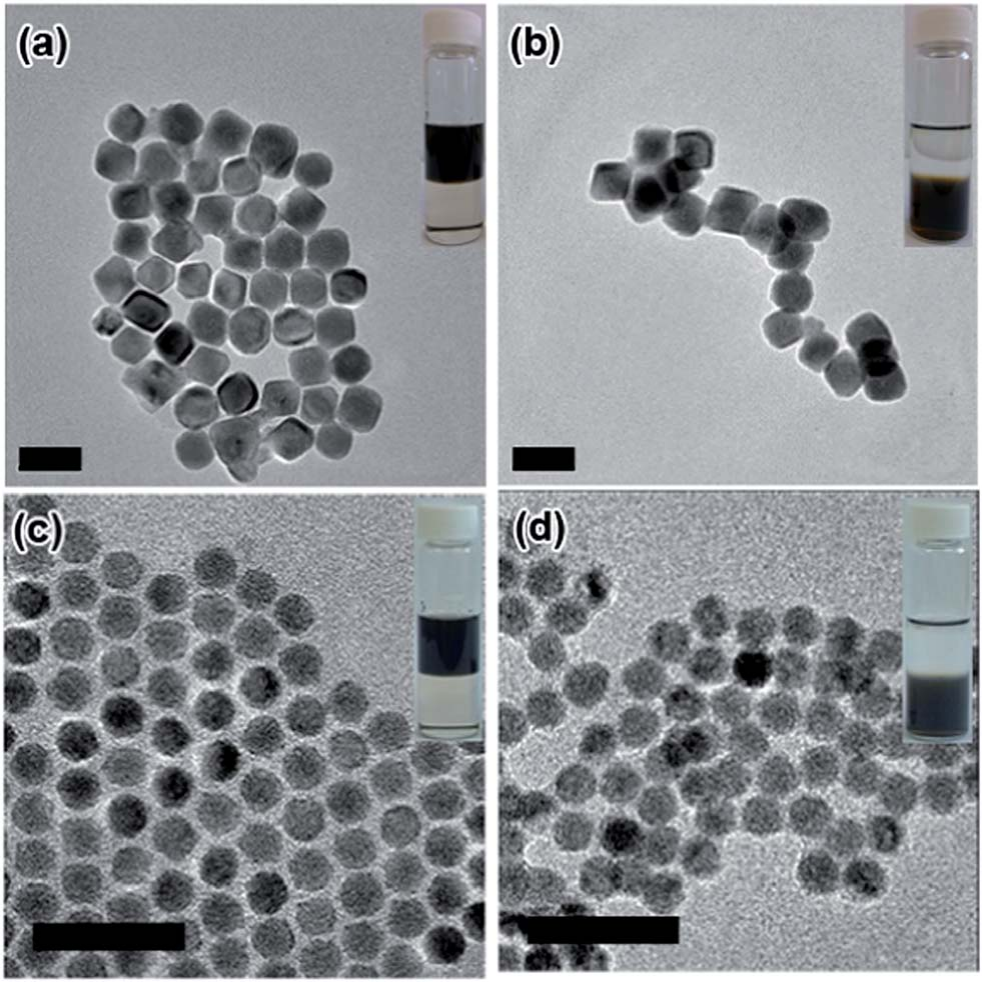
TEM images of Pb-based NPs before (a and c) and after (b and d) GQD ligand exchange. (a and b) PbTe, 30 nm; (c and d) PbSe, 10 nm. The scale bars are 50 nm. Insets are photos of NPs dispersed in the (top) non-polar phase, toluene, and (bottom) the polar phase, formamide, with GQDs.

The insets in Fig. 2 and S1† present photographs of the NPs transferring from the non-polar phase (top phase) into the polar phase (bottom phase) with aid of the GQDs. The transfer starts upon contact of the GQD-containing polar phase with the NPs in toluene. Pb-based NPs are readily transferred within hours, while Cd-based NPs require a slightly longer time to accomplish the transfer, demonstrating the universality of GQDs as a phase transfer agent. This difference could be attributed to the different affinities of the surface cations (Pb *vs.* Cd) with GQDs. The TEM images before and after phase transfer indicate that the NPs’ shape and size are preserved (Fig. 2). A thermogravimetric analysis shows that, as for 18 nm PbTe NPs, weight loss is 17% before GQD exchange and 8% after GQD exchange (Fig. S2†). By comparing the final residue weight of GQD–PbTe nanocomposites and that of pure GQDs, we estimate that the weight percentage of GQDs in this composite is about 14%.

**Fig. 3:**
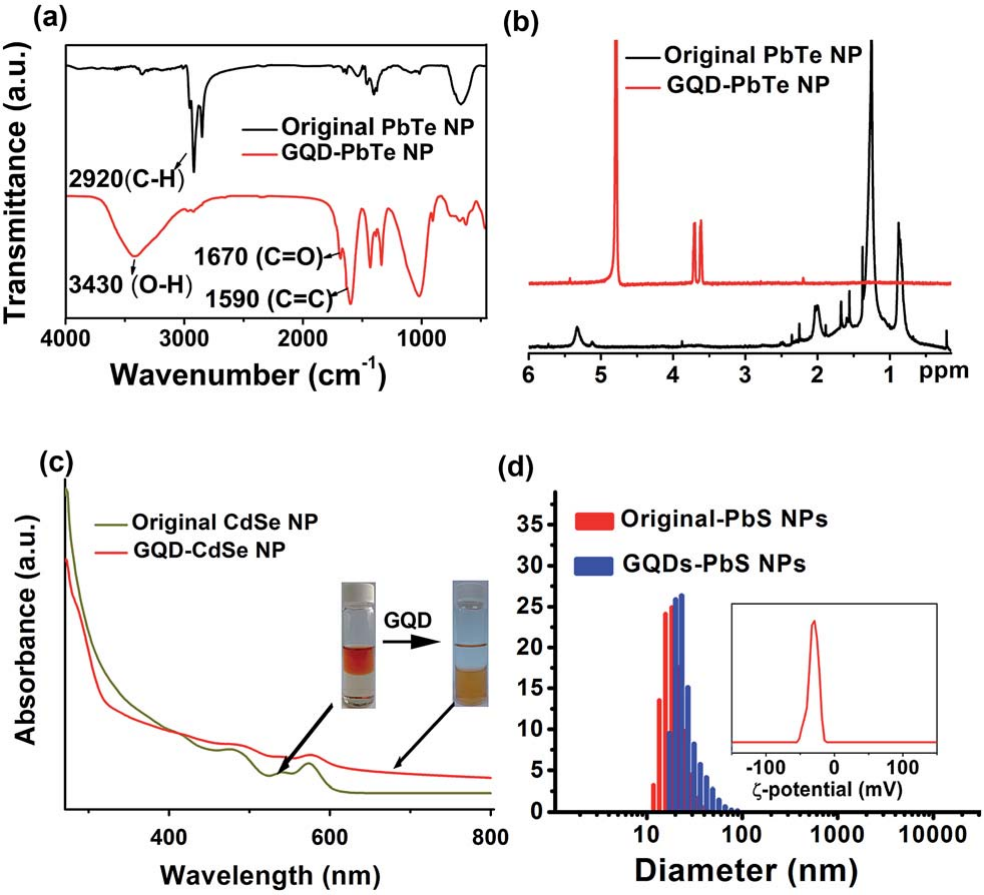
(a) FTIR spectra and (b) ^1^H NMR spectra of PbTe NPs before and after ligand exchange; (c) UV-vis spectra of CdSe NPs before and after ligand exchange. The first exciton peak of the NPs is preserved, while the GQD absorption feature near 287 nm is additionally observed. This indicates that the NP core structures are preserved during ligand exchange. (d) Size distribution of PbS NPs before (red) and after (blue) GQD ligand exchange; the inset shows the *ζ*-potential after GQD ligand exchange.

The Fourier transform infrared spectroscopy (FTIR) spectrum of the NP dispersion after phase transfer shows a greatly reduced peak for the C–H stretching mode at ∼2900 cm^–1^ (Fig. 3a and S3†), confirming the removal of alkyl chains on the surface of the NPs. The peaks at 1670 cm^–1^ and 1590 cm^–1^ are assigned to the stretching modes of C=O and C=C, respectively, indicating the presence of GQDs after ligand exchange. Furthermore, ^1^H nuclear magnetic resonance (NMR) spectroscopy clearly proves the exclusive removal of alkyl H atoms in the range of 0.8–2.5 ppm and alkene H atoms at 5.3 ppm (Fig. 3b and S4†); the chemical shifts at 3.6 ppm and 3.7 ppm of the GQD–NPs are assigned to the two types of H atoms on GQDs (Fig. S4†). This spectroscopic evidence is consistent with the proposed scheme (Fig. 1a) in which the native ligands of NPs are totally exchanged by GQDs after phase transfer.

X-ray diffraction (XRD) patterns for PbTe NPs before and after GQD ligand exchange are shown in Fig. S5a.† Evidently, there is no observable change in the structural integrity for NPs during the phase transfer process. UV-vis spectroscopy of CdSe NPs before and after GQD ligand exchange further reveals no obvious shifting in the first exciton peak (Fig. 3c), and HRTEM imaging (Fig. S6†) also suggests no modification of the NP cores occurs upon GQD-coating.

It would be interesting to learn how the GQDs assemble near the surface of the NPs to form a stable dispersion in a polar solvent. We therefore performed a dynamic light scattering (DLS) study to reveal this behaviour *in situ*. Fig. 3d shows that the GQD–PbS NP complexes are dispersed in DMSO with a uniform hydrodynamic diameter slightly larger than that of the original NPs with organic ligands. This could be explained by the larger size of the solvation shells containing GQDs and polar solvent molecules compared to those containing the native oleic acid ligands. It should be noted that such shells collapse due to loss of solvent in the TEM chamber, resulting in the closer inter-NP distance shown in Fig. 2. All these complexes formed stable colloidal solutions and were stored at ambient conditions for more than 3 months without noticeable changes. The negatively charged surfaces (the negative *ζ*-potential in the insets of Fig. 3d and S5b†) suggest that the GQDs are grafted onto NP surfaces with some of their carboxylic groups, while the remaining carboxylic groups facing the polar solvent are deprotonated thus stabilizing the solvated NPs *via* increasing their energy of solvation. The proposed scenario is presented in Fig. 1a. It should be noted that the small size of GQDs limits us from obtaining more *in situ* details of their status in the GQD–NP complexes, which still remains a major challenge for almost all types of ligands on NP surfaces.

**Fig. 4:**
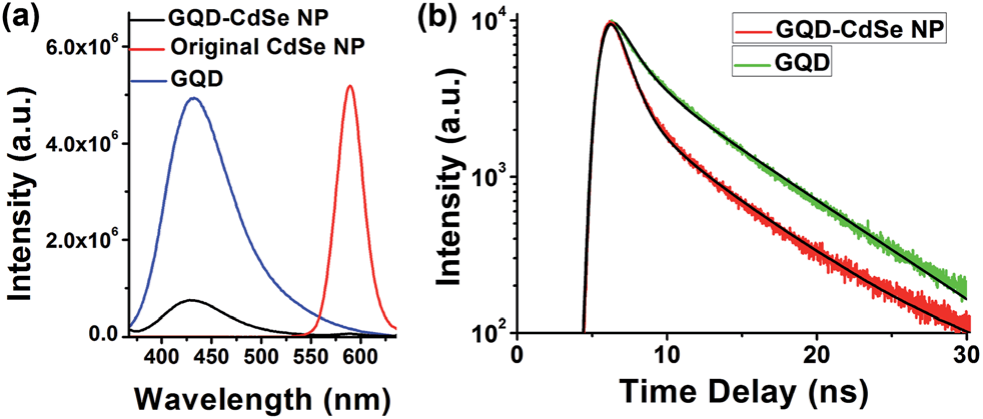
(a) PL of GQDs alone (blue), CdSe NPs alone (red), and GQDs capping CdSe NPs (black), showing dramatically quenched emission for both CdSe NPs and GQDs in the composite. (b) Transient spectroscopy of GQD PL emission. For the two major decay branches, the lifetimes of GQDs alone are *τ*_1_ = 1.34 ns and *τ*_2_ = 6.77 ns, while the lifetimes of CdSe NPs with GQDs ligands are *τ*_1_ = 0.98 ns and *τ*_1_ = 6.56 ns.

To further prove the binding of GQDs to NPs, we studied the PL of GQD-capped CdSe NPs dispersed in solution (Fig. 4).^43^,^44^ CdSe NPs were selected because their emission is located in the visible range and easily measured. The PL intensity of GQDs after binding with the NPs is decreased by ∼90% (Fig. 4a), and the lifetimes for the two major decay branches are reduced from *τ*_1_ = 1.34 ns and *τ*_2_ = 6.77 ns to *τ*_1_ = 0.98 ns and *τ*_2_ = 6.56 ns, respectively (Fig. 4b), which suggests the occurrence of short distance energy transfer from GQDs to CdSe NP cores. Meanwhile, the emission from CdSe NPs is almost completely quenched. We compared high resolution TEM images (Fig. S6†) for CdSe NPs before and after GQD ligand exchange and found no obvious change in their crystallinity, which indicates that the PL quenching of CdSe NPs is caused by poor surface trap passivation instead of core structural change. We suppose that passivation of CdSe NP surface traps with wide-band-gap shells such as ZnS would preserve the PL characteristics of CdSe NPs and allow us to better elucidate the energy transfer between GQDs and CdSe NPs. Such a detailed study is underway in our group to gain a deeper understanding of the energy flow in this composite material. The spectroscopy results indicate that GQDs are bound to the surface of the NPs and serve as ligands instead of free floating in the solution. Such interaction and energy transfer between GQD ligands and NP cores might also permit engineering of versatile properties into this type of composite, enabling progress toward functional materials.

**Fig. 5:**
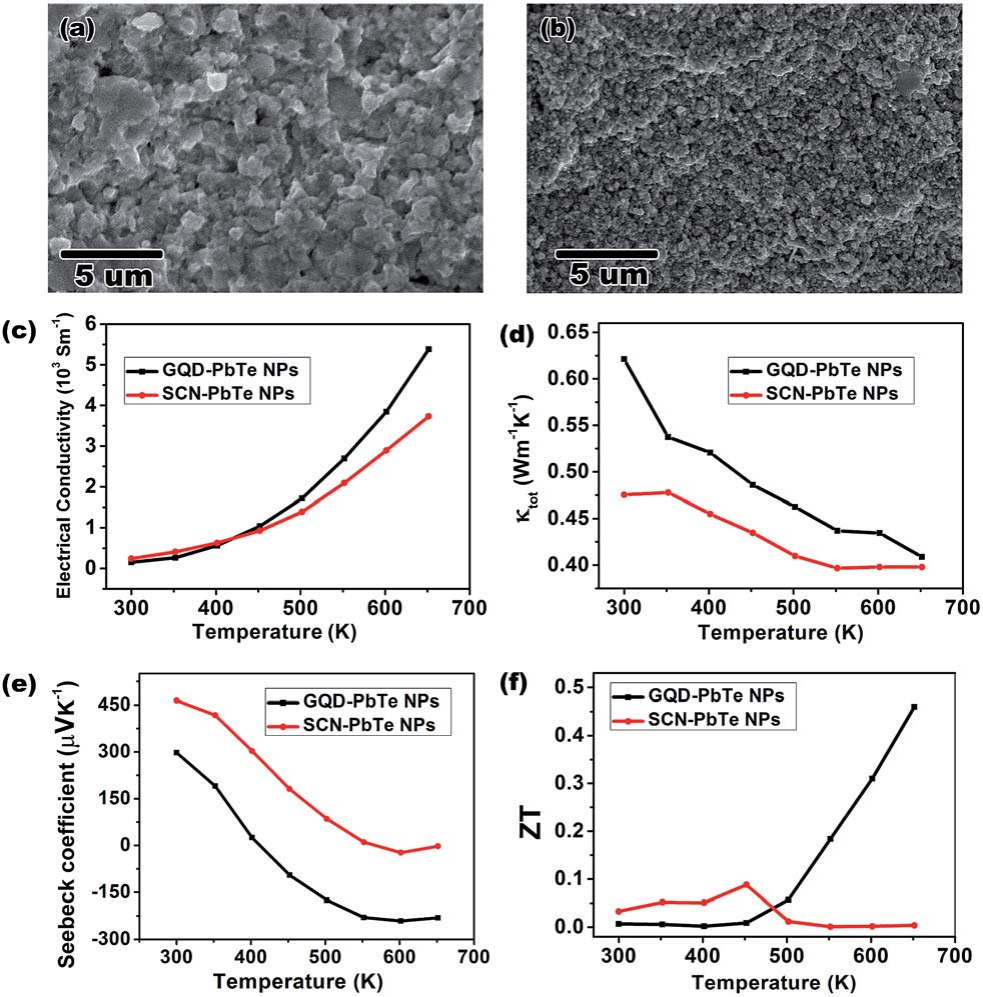
The cross-section SEM images of pellets of SCN–PbTe NPs, (a), and GQD–PbTe NPs, (b) by SPS. The measured electrical conductivity, (c), thermal conductivity, (d), Seebeck coefficient, (e), and calculated ZT values, (f), are presented as functions of temperature.

Composites made from NPs are promising materials for thermoelectric applications, because of their inherent low thermal conductivity and enhanced Seebeck coefficients that result from quantum confinement and energy filtering effects.^35^ However, NPs generally suffer from alloying and fusing at elevated temperatures, which weakens these effects and leads to an irreversibly decreased Seebeck coefficient.^45^,^46^ By capping the PbTe NPs with GQDs, we demonstrate that thermally stable GQDs effectively lessen sintering of NPs in the composite. We made pellets (Fig. S7†) of GQD-capped (GQDs from coal oxidation, see ESI†) PbTe NPs by SPS for thermoelectric measurements. For comparison, control pellets were also prepared using PbTe NPs capped by the most common ligands, SCN anions, which decompose into gaseous species at the SPS temperature of 450 °C (ref. 3 and 47). Scanning electron microscopy (SEM) images of the cross sections of these two pellets reveal that much finer nanostructures exist in the pellets of GQD-capped NPs than those in SCN-capped NPs (Fig. 5a and b). The X-ray diffraction pattern also confirms that the PbTe crystalline domain size is smaller in the former composite (Fig. S8 and Table S1†). The presence of GQDs after SPS was confirmed by Raman spectroscopy (spectrum shown in Fig. S7b†), indicating its thermal stability. The above observations suggest that GQD-capping around PbTe NPs lessens their propensity to fuse together and maintains smaller crystalline domains. Such an effect leads to beneficial thermoelectric properties with enhanced Seebeck coefficients resulting from nanostructuring.

The thermoelectric measurements highlight the advantages of using GQDs over SCNs as capping agents for both electrical conductivity and Seebeck coefficient (Fig. 5c–f). The final calculated figure of merit (ZT) shows a peak value of 0.46 at 650 K for the GQD–PbTe NP complex, which is among the best for solution processed pure PbTe thermoelectric materials.^48^–^50^ Numerically, the main reason for the good ZT is attributed to the enhanced n-type Seebeck coefficient at 650 K. The switching of conduction type from p to n for both pellets can be understood as the excitation of electrons in the composite at elevated temperature. Interestingly, the GQD-capped NP complex shows a much quicker conduction-type switch and has a higher Seebeck coefficient value when a plateau is reached (Fig. 5e). The mechanisms to account for such enhanced Seebeck coefficient may be complicated. Besides the aforementioned quantum confinement effect, both electron doping and the carrier filtering effect of GQDs may also play a role, since a heterojunction is formed between the GQDs and the PbTe matrix. More detailed studies, therefore, are needed to understand the thermoelectric behaviour of the GQD–PbTe composite. Nevertheless, the GQD-capped NP complex shows a considerable ZT value even without tuning of composition and doping of the NPs,^51^ which suggests the composite is a good candidate material for thermoelectric device fabrication. Given the thermal stability of GQDs, it is also advantageous over molecular capping ligands for controlling crystal size for optimized thermoelectric properties.

The prepared GQD–NP composite may also be applied when the collective properties of different components are desired. We have demonstrated the effect of GQDs in lessening the sintering of PbTe NPs for enhanced thermoelectric performance. Another application is likely to be photovoltaic materials. There are reports of mixing GQDs with TiO_2_ NPs^44^,^52^ or ZnO nanowires^53^ to improve solar cell performance by taking advantage of energy transfer between the GQDs and other nanomaterials. The tunable band structure of GQDs, together with their good interfacing with NPs, allows one to fine tune such energy transfer between GQDs and NPs in the hope of yielding a composite with novel properties, again superior to molecular ligands. Recent progress on large scale GQD production^26^,^54^,^55^ will permit mass production of these GQD–NP composites for industrial applications.

## Conclusions

In conclusion, for the first time, we reported the general capability of GQDs to serve as capping ligands exchanging native organic stabilizers for various types of semiconductor NPs. The FTIR, NMR, TEM and XRD characterization results proved that the ligand exchange is complete and that the integrity of the NPs is preserved. Thermoelectric measurement of GQD–PbTe composites revealed that the GQDs play a crucial role in limiting crystal size leading to an enhanced Seebeck coefficient, and thus a considerable ZT value of 0.46, without tuning the composition or doping level of the NPs. The PL lifetime of the GQD–NPs indicated efficient energy transfer between the GQD ligands and the NP cores. Given the many and yet tunable properties of GQDs, we anticipate that versatile properties could be engineered from this novel type of GQD–NP composite and to benefit various applications, including photovoltaic and thermoelectric devices, and catalysis.

## Supplementary Material

Supplementary information
