# Supplementary material for: Capping nanoparticles with graphene quantum dots for enhanced thermoelectric performance
Source: Chem Sci. 2015 Apr 13;6(7):4103–8. doi: 10.1039/c5sc00910c (PMC5497257; doi:10.1039/c5sc00910c)
Supplement: Supplementary file 1 [file SC-006-C5SC00910C-s001.pdf]

Electronic Supplementary Material (ESI) for Chemical Science.
This journal is © The Royal Society of Chemistry 2015

Supporting Information

Capping Nanoparticles with Graphene Quantum Dots for Enhanced Thermoelectric Performance

Yuantong Liang[1,2], Chenguang Lu[1], Defang Ding[1], Man Zhao[1], Dawei Wang[1], Chao Hu[3], Jie-shan Qiu[3], Gang Xie[2*] , Zhiyong Tang[1*]*

Nanoparticle (NP) synthesis:

PbSe NPs: PbSe NPs were synthesized using a modified literature method[S[1]]. In a typical reaction, 1.08 g of lead acetate trihydrate, 2.7 mL of oleic acid and 20 mL of diphenyl ether were dehydrated under vacuum at 100°C for 1 h. Then the temperature was increased to 180°C under nitrogen and 9 mL of 1 M trioctylphosphine selenide (TOPSe) in trioctylphosphine (TOP) solution was injected into the solution. The solution was maintained at 150~155 °C for 5 min. The reaction solution was cooled to room temperature and washed twice with ethanol/toluene, and redispersed in toluene.

PbS NPs: PbS NPs were made according to a literature method[S[2]]. In a typical process, 0.18 g of lead oxide, 8 mL of octadecene (ODE) and 12 mL of oleic acid were added to a 50 mL three-neck flask. The mixture solution was dried under vacuum at 100°C for 1 h. The solution was heated to 120°C before injection. Then 4 mL of 0.1 M bis(trimethylsilyl) sulfide was injected into the reaction mixture, which was maintained at 105°C for 30 min. The reaction solution was washed twice using ethanol when the temperature was cooled to room temperature and re-dispersed in toluene.

CdSe NP: CdSe NPs were made according to a literature method[S[3]]. The CdSe NPs were synthesized by dissolving 0.051 g cadmium oxide in 15 mL ODE containing 0.455 g stearic acid, and then 2 g trioctylphosphine oxide and 2 g hexadecylamine were added. The solution was

dried under vacuum for 1 h at 100°C. The solution was then heated to 300°C, and 2 mL of 1 M TOPSe in TOP was injected. The temperature was maintained at 280°C for 30 min for NP growth. The product was washing three times using ethanol/toluene and re-dispersed in toluene.

GQD preparation:

From graphite: Graphene oxide (GO) was generated from natural graphite powder by a modified Hummer's method[S[4]]. Graphene quantum dots (GQDs) were prepared according to literature methods[S[5]]. GO (1 g) was oxidized by sonicating in concentrated H2SO4 and HNO3 for 10 h. The mixture was then diluted with deionized water and filtered through 0.22 μm filter to remove the acids. The GO was re-dispersed in pure water and the pH was adjusted to 8 with NaOH solution. The suspension was transferred to a poly(tetrafluoroethylene) (PTFE)-lined autoclave (200 mL) and heated at 200°C for 10 h. After cooling to room temperature, the resulting black suspension was filtered through a 0.22 μm membrane and a brown filtration solution was collected. The colloidal solution was dialyzed in a 3500 Da dialysis bag for 4 days and again in a 500 Da dialysis bag for 36 h. The GQDs solution was dried by a vacuum rotary evaporator and re-dissolved in formamide.

From coal: The coal sample (Chinese anthracite coal from Taixi mine) was first crushed and sieved to 200 μm, and then carbonized at 900°C for 2h in a N_2 atmosphere. Afterwards, the coal derivative coke was put into nitric acid (70 mL, 6 M), treated under ultrasonic conditions for 2 h, and then heated at 140°C for 24 h. After that, the mixture was cooled back to room temperature naturally, and sodium carbonate was added to adjust its pH to 7, yielding supernatant yellow colloidal that was separated from the sediment by centrifuging at 9000 rpm for 30 min, and then dialyzed (3500 Da) for 2 days to remove the nitrate.

Materials and Characterization:

Materials:

Tellurium powder (99.99%), selenium powder (99.99%), selenium powder (99.99%), oleic acid (90%), diphenyl ether (90%), octadecene (90%), bis(trimethylsilyl) sulfide, hexadecylamine, and tricotylphosphine oxide (98%) were purchased from Alfa Aesar. Tricotylphosphine was purchased from StremChem. Lead oxide (99.999%), nitric acid, sulfuric acid, sodium hydroxide, lead acetate trihydrate, cadmium oxide, and natural graphite were supplied by Sinopharm. All chemicals were used without further purification.

Characterization tools:

Transmission electron microscopy (TEM) was performed on Tecnai G^2 F20 S-TWIN TEM at 200kV. Scanning electron microscopy (SEM) images were obtained with a Hitachi S4800 scanning electron microscope at 30 kV. Powder X-ray diffraction (XRD) patterns were recorded with D/MAX-TTRIII (CBO) with Cu Kα radiation (λ = 1.542 Å) operating at 50 KV and 300 mA. Fourier transform infrared (FTIR) spectra were recorded on Spectrum One in spectral range of 400-4000 cm^{-1} using the KBr disk method. Thermogravimetric analysis (TGA) was collected using a PerkinElmer Diamond TG/TGA under nitrogen flow at a heating rate of 5°C/min. ^1H NMR spectra was measured by Bruker Advance 400 spectrometer (400 MHz). Photoluminescence and its lifetime were characterized by Varian PL-GPC•50 and Horiba Jobin Yvon Nanolog system. Raman spectrum was recorded with Renishaw inVia Plus.

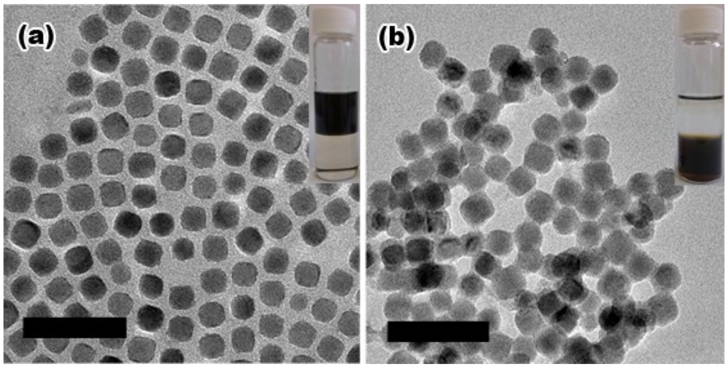

Figure S1: TEM images of PbS NPs before (a) and after (b) GQD ligand exchange. The scale bars are 50 nm.

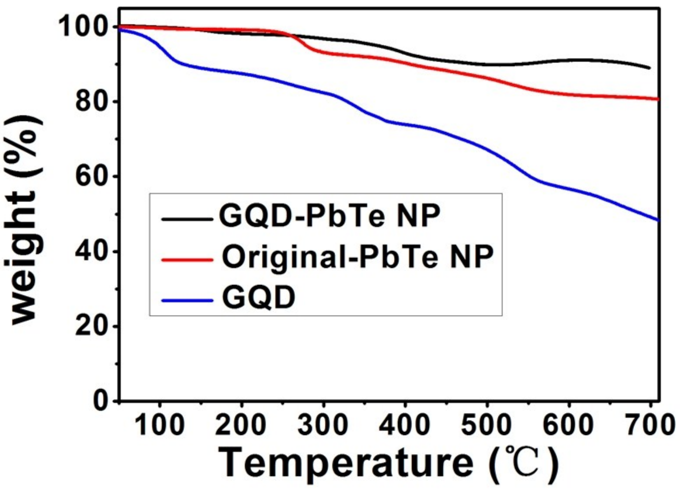

Figure S2: TGA curves of PbTe (18 nm) NPs before (blue) and after (black) GQD ligand exchange, and pure GQD (red). The weight loss is 8% for PbTe NPs after ligand exchange and 46% for pure GQD.

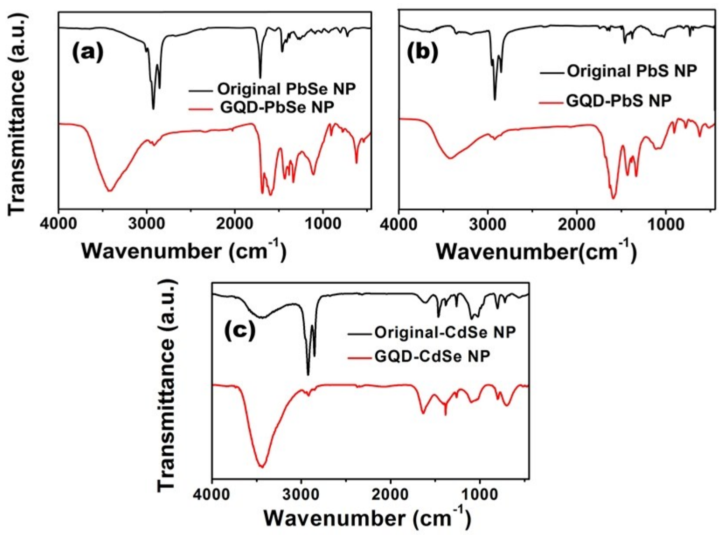

Figure S3: a), b), and c) FTIR spectra of PbSe NPs, PbS NPs, and CdSe NPs before and after ligand exchange using GQDs, respectively. The characteristic C-H stretching of original ligand oleic acid at 2900 cm^{-1} disappears after ligand exchange, confirming the removal of alkyl chains on the surface of NPs. The peaks at 1670 cm^{-1} and 1590 cm^{-1} after ligand exchange are assigned to stretch mode for C=O and C=C, respectively, indicating GQD's presence after ligand exchange.

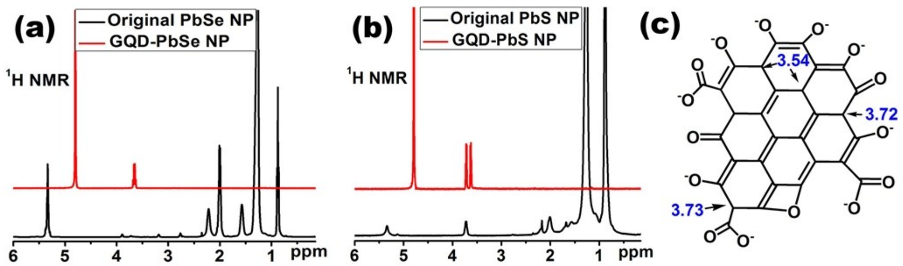

Figure S4: ^{1}H NMR spectra of a) PbSe NPs and b) PbS NPs before and after GQD ligand exchange. c) Peaks near 3.5 ppm and 3.7 ppm are confirmed by simulation of graphene structure using computer software to be H atoms with different chemical environment.

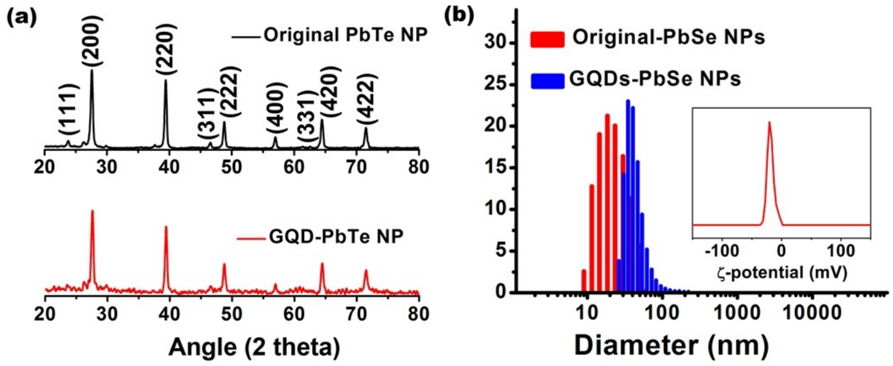

Figure S5: a) XRD patterns of PbTe NPs before and after ligand exchange; b) Size distributions of various NPs before (red) and after (blue) GQD ligand exchange based on dynamic light scattering measurement. Inset is their ζ-potential after GQD ligand exchange. For GQD-PbSe NP complexes shown in Figure S5b, the DLS diameter suggests a slight aggregation.

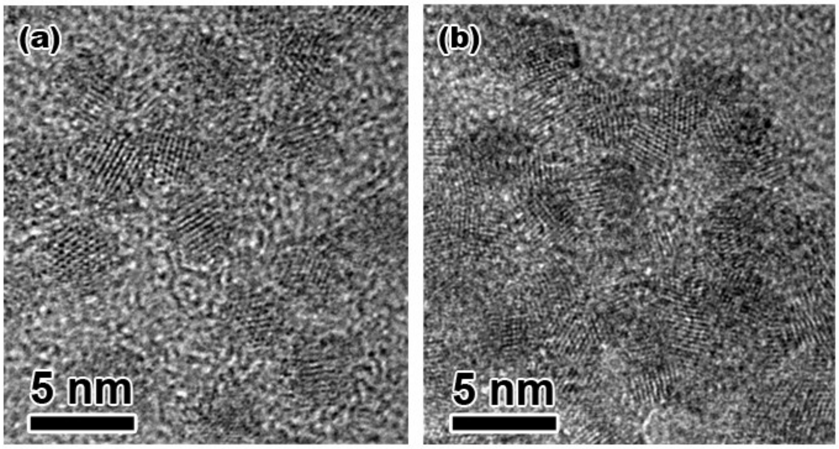

Figure S6: High resolution TEM images of CdSe NPs: a) before and b) after GQD ligand exchange.

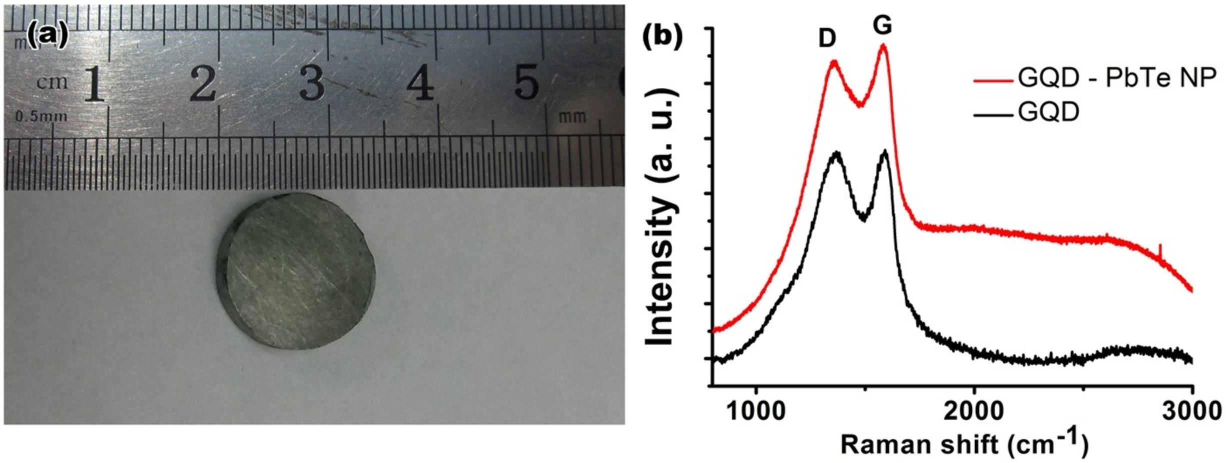

Figure S7: a) Photograph of a pellet from GQDs-PbTe NPs made by SPS. b) Raman spectrum of a pellet of GQD-PbTe NPs(red) and original GQDs(black), excited with 633 nm laser. G and D peaks indicate presence of GQDs before and after SPS treatment.

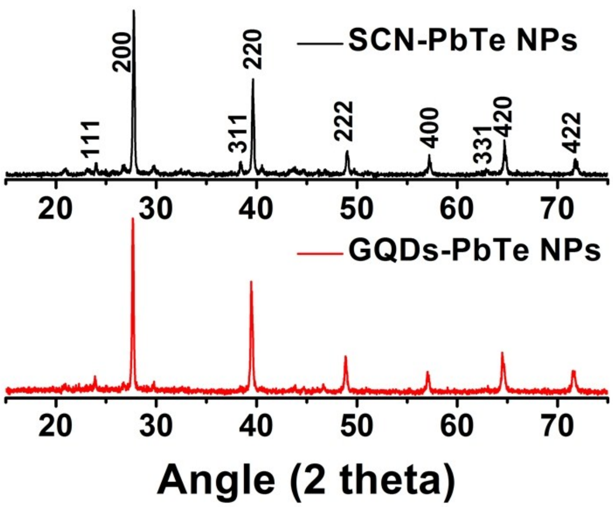

Figure S8: XRD Patterns of pellets made from SCNs or GQDs capped PbTe NPs.

Table S1: Crystalline sizes of PbTe NPs in the SPS pellets, fitted by Scherrer equation using Jade 5.0 XRD software.

	Peak*	200	220	222	400	420	422	average (nm)
Crystalline sizes (nm)	SCNs-PbTe NPs	43.3	43.9	40.1	42.	46..9	37.0	42.28
	GQDs-PbTe NPs	42.0	34.2	34.7	34.3	35.6	27.4	34.56

S[1] K.-S. Cho, D. V. Talapin, W. Gaschler, C. B. Murray, *J. Am. Chem. Soc.* **2005**, *127*, 7140.

S[2] M. A. Hines, G. D. Scholes, *Adv. Mater.* **2003**, *15*, 1844.

S[3] Z. A. Peng, X. Peng, *J. Am. Chem. Soc.* **2000**, *123*, 183.

S[4] Y. Xu, H. Bai, G. Lu, C. Li, G. Shi, *J. Am. Chem. Soc.* **2008**, *130*, 5856.

S[5] D. Pan, J. Zhang, Z. Li, M. Wu, *Adv. Mater.* **2010**, *22*, 734.
